# Physical activity in pregnancy: a Norwegian-Swedish mother-child birth cohort study

**DOI:** 10.1016/j.xagr.2020.100002

**Published:** 2021-01-27

**Authors:** Oda C.L. Carlsen, Hrefna K. Gudmundsdóttir, Karen Eline S. Bains, Randi Bertelsen, Karin C.L. Carlsen, Kai-Håkon Carlsen, Kim M.A. Endre, Berit Granum, Guttorm Haugen, Gunilla Hedlin, Christine M. Jonassen, Ina Kreyberg, Linn Landrø, Caroline-Aleksi Olsson Mägi, Björn Nordlund, Live S. Nordhagen, Kristian Pehrson, Carina M. Saunders, Katrine Sjøborg, Håvard O. Skjerven, Anne Cathrine Staff, Cecilie Svanes, Cilla Söderhäll, Riyas Vettukattil, Magdalena Værnesbranden, Johanna Wiik, Eva Maria Rehbinder

**Affiliations:** a Department of Global Public Health and Primary Care, University of Bergen, Bergen, Norway (Ms O Carlsen and Dr Svanes); b Division of Paediatric and Adolescent Medicine, Oslo University Hospital, Oslo, Norway (Drs Gudmundsdóttir, Bains, K Carlsen, Kreyberg, Saunders, and Skjerven); c Faculty of Medicine, Institute of Clinical Medicine, University of Oslo, Oslo, Norway (Drs Gudmundsdóttir, Bains, KC Carlsen, K-H Carlsen, Endre, Haugen, Kreyberg, and Landrø, Ms Nordhagen, and Drs Saunders, Skjerven, Staff, Vettukattil, Værnesbranden, and Rehbinder); d Department of Clinical Science, University of Bergen, Bergen, Norway (Ms Bertelsen); e Oral Health Centre of Expertise in Western Norway, Hordaland, Bergen, Norway (Ms Bertelsen); f Department of Dermatology and Venerology, Oslo University Hospital, Oslo, Norway (Drs Endre, Landrø, and Rehbinder); g Department of Environmental Health, Norwegian Institute of Public Health, Oslo, Norway (Dr Granum); h Division of Obstetrics and Gynaecology, Oslo University Hospital, Oslo, Norway (Drs Haugen and Staff); i Astrid Lindgren Children's Hospital, Karolinska University Hospital, Stockholm, Sweden (Dr Hedlin, Ms Mägi, and Drs Nordlund and Söderhäll); j Department of Women's and Children's Health, Karolinska Institute, Stockholm, Sweden (Dr Hedlin, Ms Mägi, and Drs Nordlund and Söderhäll); k Genetic Unit, Centre for Laboratory Medicine, Østfold Hospital Trust, Kalnes, Norway (Dr Jonassen); l Faculty of Chemistry, Biotechnology and Food Science, Norwegian University of Life Sciences, Ås, Norway (Dr Jonassen); m VID Specialized University, Oslo, Norway (Ms Nordhagen); n Faculty of Mathematics and Natural Sciences, University of Oslo, Norway (Pehrson); o Department of Obstetrics and Gynecology, Østfold Hospital Trust, Kalnes, Norway (Drs Sjøborg, Værnesbranden, and Wiik); p Department of Obstetrics and Gynecology, Institute of Clinical Sciences, Sahlgrenska Academy, Gothenburg University, Gothenburg, Sweden (Dr Wiik)

**Keywords:** bicycling, brisk walking, maternal exercise, maternal health, mother-child birth cohort, physical activity, pregnancy, risk factors, strength training

## Abstract

**BACKGROUND:**

Physical activity during pregnancy is important for maternal and offspring health. Optimal conditions during pregnancy may help reduce the burden of noncommunicable diseases. National and international guidelines recommend at least 150 minutes of physical activity of at least moderate intensity per week. To optimize physical activity in pregnant women, it is important to identify factors associated with higher levels of physical activity.

**OBJECTIVE:**

This study aimed to explore types and levels of physical activity in midpregnancy in Norway and Sweden and to identify factors associated with higher levels of physical activity.

**MATERIALS AND METHODS:**

From the population-based mother-child cohort Preventing Atopic Dermatitis and Allergies in Children study recruiting 2697 women in Norway and Sweden from 2014 to 2016, we included 2349 women who answered an electronic questionnaire at enrollment in midpregnancy. Women were asked about regular physical activity in the last 2 weeks of pregnancy and afterward for types and levels of physical activity in pregnancy and before pregnancy and socioeconomic status, lifestyle, and maternal health. Logistic regression analyses were used to identify factors associated with higher levels of physical activity in pregnancy, defined as >30 minutes per session of ≥2 times per week of moderate- or high-intensity brisk walking, strength training, jogging, and bicycling.

**RESULTS:**

No regular physical activity during the last 2 weeks before answering the questionnaire at midpregnancy was reported by 689 women (29%). In this study, 1787 women (76%) reported weekly strolling during pregnancy. Regular physical activity at least twice weekly in the first half of pregnancy was reported as brisk walking by 839 women (36%), bicycling by 361 women (15%), strength training by 322 women (14%), and other activities by <10% of women. Among the 1430 women with regular moderate- or high-intensity physical activity, the estimated median duration per week was 120 minutes. Higher physical activity levels were achieved in 553 women (23.5%) by brisk walking, 287 women (12.2%) by strength training, 263 women (11.2%) by bicycling, and 114 women (4.9%) by jogging. Higher physical activity levels were positively associated with regular physical activity before pregnancy, dog ownership, and atopic dermatitis and negatively associated with higher body mass index, study location in Østfold, previous pregnancy or pregnancies, non-Nordic origin, suburban living, and sick leave.

**CONCLUSION:**

At midpregnancy, 29% of women were inactive, and less than 50% of women had at least 2 hours of moderate-intensity physical activity weekly. Awareness of physical activity in pregnancy should be discussed at pregnancy follow-up visits, particularly among women with higher body mass index, sick leave, previous pregnancy or pregnancies, and non-Nordic origin.

**AJOG MFM at a Glance**

**Why was this study conducted?**

Physical activity (PA) in pregnancy is important for maternal and offspring health. This study aimed to explore types and levels of midpregnancy PA and factors associated with higher PA levels in women participating in a Scandinavian mother-child birth cohort.

**Key findings**

Strolling, brisk walking, strength training, and bicycling were most commonly performed at midpregnancy; furthermore, 29% reported no PA in the last 2 weeks before inclusion. Less than 50% reported 120 minutes or more of at least moderate-intensity PA per week. Higher levels of PA were significantly associated (*P*-value <.05) with regular PA before pregnancy and dog ownership and negatively associated with higher body mass index, previous pregnancy or pregnancies, non-Nordic origin, suburban living, and sick leave.

**What does this add to what is known?**

In this recently established mother-child birth cohort, most women performed less than 2 hours of moderate-intensity PA weekly. Our study points to the need to discuss PA during pregnancy follow-up visits.

Optimal conditions during pregnancy may reduce the burden of noncommunicable diseases in the offspring,^1^, ^2^, ^3^ and modifiable lifestyle factors during pregnancy have been associated with maternal and offspring health. Physical activity (PA) in pregnancy is considered beneficial and safe for the mother and fetus^3^, ^4^, ^5^, ^6^ and seems to reduce the risk of cesarean delivery,^7^^,^^8^ hyperemesis gravidarum,^9^ gestational diabetes mellitus, hypertensive disorders of pregnancy, excessive gestational weight gain, lumbopelvic pain, and preterm birth.^5^^,^^10^ Avoiding these adverse pregnancy outcomes seemed to have benefits in women's future health.^11^, ^12^, ^13^, ^14^, ^15^

Patterns of PA among pregnant women have been previously studied^16^, ^17^, ^18^, ^19^; however, this knowledge needs to be regularly updated to continually enhance maternity care. Brisk walking and swimming were the most commonly performed exercises in the Avon Longitudinal Study of Parents and Children (ALSPAC)^16^; however, a recent Danish cross-sectional study found that bicycling was the most prevalent activity, followed by brisk walking.^17^

Recommendations for PA during pregnancy are available in many countries.^20^ To improve health-related outcomes, pregnant women should perform at least 150 minutes of moderate- to high-intensity exercise per week.^3^^,^^5^ These recommendations were implemented in Norway in 2019.^21^ Nonpregnant adults have similar recommendations, with the addition that ≥150 minutes of moderate-intensity PA may be replaced with ≥75 minutes of high-intensity PA.^22^

To optimize PA in pregnancy, it is important to identify factors associated with lower and higher levels of PA. Primiparity and normal weight have been shown to increase the likelihood of higher PA levels in Scandinavian studies,^17^^,^^18^ whereas in United Kingdom studies, dog ownership has been associated with more regular PA in pregnancy.^23^ Several other factors have previously been explored, such as maternal age, education, regular PA before pregnancy, body mass index (BMI), and smoking, with differing results.^19^

In this study, the primary aim was to explore the types and levels of PA reported in midpregnancy in Norway and Sweden, and the secondary aim was to identify factors associated with higher levels of the most commonly performed physical activities in pregnancy.

## Materials and Methods

### Study design

Data from the Preventing Atopic Dermatitis and Allergies in Children (PreventADALL) study,^24^ a Scandinavian general population-based mother-child birth cohort, enrolling 2697 women from December 2014 to October 2016, were used in this substudy on PA in pregnancy.

Pregnant women were recruited during the routinely offered 18 weeks’ gestation ultrasound examination and enrolled in the PreventADALL study at Oslo University Hospital, Østfold Hospital Trust, Norway, and Karolinska University Hospital, Stockholm, Sweden. All women attending the 18 weeks’ gestation routine ultrasound examination at 1 of the participating facilities were invited to participate by letter of invitation attached to the appointment letter and information about the study by the midwife or the study personnel at the maternity clinic. After the ultrasound examination, women were invited to the study facility where they received further information from the study team before enrollment.

The inclusion criteria for the PreventADALL study were gestational age (GA) of 16 to 22 weeks at the time of the routine ultrasound examination, singleton or twin pregnancy, no severe fetal disease, and proficiency in the Scandinavian language.

At the enrollment visit, all women signed informed consent forms, followed by a brief interview; measurements of weight, height, and blood pressure; recording of ultrasound examination data; and information about study participation. The women were asked to complete a detailed electronic questionnaire (e-questionnaire) shortly after enrollment,^24^ which provided the basis for this study. The e-questionnaire was sent by email, followed by 1 reminder the following week if there was no response, ensuring 1 response only.

### Study population

In this study, we included 2349 women (87%) who returned the e-questionnaire associated with enrollment. The 351 nonresponding women (13%) were similar to those included in the study in age, parity, and BMI (Table 1).

**Table 1:** Background characteristics of the 2349 respondents and the 351 nonrespondents of the 18-week electronic questionnaire in the PreventADALL study

Characteristics	Respondents (present study participants) (n=2349)	Nonrespondents (excluded from this study) (n=351)
Age (y)	n=2349 32.4 (4.1)	n=351 31.8 (4.7)
Prepregnancy weight (kg)	n=2296 65.4 (11.3)	n=338 66.0 (12.4)
Weight gain at 18 wk of pregnancy (kg)	n=2293 4.7 (3.2)	n=336 4.3 (3.2)
Weight at 18 wk of pregnancy (kg)	n=2321 70.2 (11.3)	n=341 70.4 (12.6)
BMI at 18 wk of pregnancy (kg/m^2^)	n=2311 24.8 (3.7)	n=341 25.0 (4.2)
Marital status		
Married or cohabitant	2280 (97.1)	
Single	44 (1.9)	—
Other	25 (1.1)	
Previous pregnancy or pregnancies		—
Yes	1292 (55.0)	
No	1057 (45.0)	
Previous delivery or deliveries		—
0	1414 (60.2)	
1	741 (31.5)	
≥2	194 (8.3)	
Education,		—
Primary school only^a^	20 (0.9)	
High school only	239 (10.2)	
Higher education of <4 y	757 (32.2)	
Higher education of ≥4 y	1257 (53.5)	
PhD	67 (2.9)	
Missing	9 (0.4)	
Family income		—
<300,000 NOK/SEK	30 (1.3)	
300,000–600,000 NOK/SEK	306 (13.0)	
600,000–1,000,000 NOK/SEK	959 (40.8)	
1,000,000–1,400,000 NOK/SEK	743 (31.6)	
>1,400,000 NOK/SEK	270 (11.5)	
Does not wish to answer	41 (1.7)	
Country of origin		—
Norway	1562 (66.5)	
Sweden	523 (22.3)	
Other Nordic countries	31 (1.3)	
Rest of the world	233 (9.9)	
Living environment		—
City, densely populated	915 (39.0)	
City, less densely populated	882 (37.5)	
Suburb	373 (15.9)	
Countryside, outside village	52 (2.2)	
Village	127 (5.4)	
Regular physical activity before pregnancy		—
Yes	1 886 (80.3)	
No	463 (19.7)	
Dog owner		—
Yes	297 (12.6)	
No	2052 (87.4)	
Cat owner		—
Yes	259 (11.0)	
No	2090 (89.0)	
Sick leave at 18 wk		—
Yes	364 (15.5)	
No	1985 (84.5)	
Doctor-diagnosed asthma		—
Yes	405 (17.2)	
No	1944 (82.8)	
Doctor-diagnosed atopic dermatitis		—
Yes	461 (19.6)	
No	1888 (80.4)	
Doctor-diagnosed allergic rhinitis		—
Yes	477 (20.3)	
No	1872 (79.7)	
Smoke in pregnancy		—
Not in pregnancy	2233 (95)	
Quit before 18 wk GA	98 (4.2)	
Smoke at 18 wk GA	18 (0.8)	
Snus in pregnancy		—
Not in pregnancy	2171 (92.4)	
Quit before 18 wk GA	165 (7.0)	
Snus at 18 wk GA	13 (0.6)	

Data are presented as mean (standard deviation) or number (percentage).*BMI*, body mass index; *GA*, gestational age; *NOK*, Norwegian Krone; *PreventADALL*, Preventing Atopic Dermatitis and Allergies in Children study; *SEK*, Swedish Krona.

Carlsen. Physical activity in midpregnancy. Am J Obstet Gynecol Glob Rep 2021.

a Two women who answered “other” have been moved to primary school only.

### Physical activity

The women were first asked if they had been regularly physically active during the last 2 weeks of pregnancy before answering the questionnaire. All subsequent questions were related to PA typically performed during the pregnancy and the average frequency for each of the following activities: strolling, brisk walking, jogging, bicycling, strength training, aerobics, skiing, ballgames, swimming, horse riding, yoga or pilates, and other types of PA. The frequency alternatives were rarely or never, 1 to 3 times a month, once a week, 2 to 3 times a week, 4 to 5 times a week, 5 to 6 times a week, every day, and more than once per day. Regular PA before pregnancy was defined as 1 or more PAs per week with a duration of at least 20 minutes. The women were asked to compare their current level of PA during the pregnancy with their PA level before pregnancy.

The exercise intensity was recorded with the question, “How intensively do you usually exercise (so far in pregnancy)?” with the following mutually exclusive categories: no sweating or shortness of breath (low intensity), sweaty and some shortness of breath (moderate intensity), or very sweaty and very heavy breathing (high intensity).

The duration of a typical PA session was reported as <30 minutes, 30 to 60 minutes, 1 to 2 hours, or more than 2 hours. The questions on intensity and duration were based on validated questions from the Akershus Birth Cohort study,^18^ a Norwegian cohort study by Haakstad et al^25^ and the Norwegian Mother and Child Cohort study.^26^ The questions were later somewhat modified by our research team during the development of the questionnaire.

Prepregnancy weight was self-reported at the enrollment visit, where current weight was measured and recorded as kilograms with 1 decimal point. Height was measured using a standardized stadiometer. Pregnancy in gestational week was estimated on the basis of fetal femur length, as previously reported.^24^

### Outcomes, definitions, and explanatory variables

For the primary aim, the outcomes were frequency, duration, and intensity of the reported types of PA. The general activity level for each woman was estimated among women who reported activity of at least moderate intensity by adding the numbers of reported PA sessions per week and multiplying by exercise duration in minutes. Strolling, being a low-intensity activity, was excluded. Because the frequency and duration of PA was reported with a range, we calculated both the minimum and maximum numbers of minutes of PA per week. The Supplemental Information section provides further details.

Higher PA levels used in the secondary aim required PA at least 2 to 3 times a week, performed with a duration of ≥30 minutes at moderate or high intensity. Women were categorized into higher PA level for each of the 4 activities most commonly reported at least twice a week: brisk walking, bicycling, strength training, and jogging.

For each of the 4 higher level PAs, we included the following possible explanatory variables in the regression model: age, prepregnancy weight, BMI and weight gain at 18 weeks of pregnancy, marital status (cohabitant and married combined into 1 category), previous pregnancy or pregnancies, education, family income, country of origin, living environment, regular PA before pregnancy, current dog and/or cat ownership, current sick leave, smoking and/or snus use in pregnancy, doctor-diagnosed asthma, doctor-diagnosed atopic dermatitis (AD), and/or doctor-diagnosed allergic rhinitis (AR).

### Statistical analysis

The descriptive results were given as percentages of women reporting the respective activities; number (n) was listed for each activity. For univariate and multivariate analyses, missing data were set to 0, assuming that missing response reflected lack of performing the relevant activity.

To identify factors associated with higher levels of the 4 most commonly performed PAs in pregnancy, we performed univariate logistic regression analysis for potential covariates, retaining all variables with global *P* value of ≤.05 and categorical *P* values of ≤.2 in the final multivariate logistic regression model. The significance level was set to 5%.

We used Stata/SE (version 14.0; StataCorp, College Station, TX) for Windows (Microsoft Corporation, Redmond, Washington, DC) IBM Statistical Product and Service Solutions Statistics, version 26 (International Business Machines Corporation, Armonk, New York), and Microsoft Excel 2016 (Microsoft Corporation, Redmond, Washington, DC) for the statistical analyses.

### Ethical approval

The PreventADALL study was approved by the Regional Committee for Medical and Health Research Ethics in Southeast Norway (2014/518) and Stockholm (2014/2242-31/4) and registered on ClinicalTrials.gov (NCT02449850).

## Results

### Background characteristics

The mean age of the 2349 women included in this study was 32.4 years, the mean BMI at inclusion was 25, most women had higher education, and approximately half of the women were nulliparous (Table 1).

### Physical activity levels

At midpregnancy, 689 women (29%) did not report any PA during the last 2 weeks of pregnancy before answering the questionnaire. The most commonly reported regular PA performed at least once per week in the pregnancy was strolling (1787 [76.1%]), followed by brisk walking (1274 [54.2%]), strength training (707 [30.1%]), and bicycling (522 [22.2%]) (Supplemental Figure 1). PA at least twice per week was reported by 1369 women (58.3%) for strolling, 839 women (35.7%) for brisk walking, 361 women (15.4%) for bicycling, 322 women (13.7%) for strength training, and 127 women (5.4%) for jogging (Supplemental Figure 1). Yoga or pilates was reported at least once weekly by 443 women (18.9%) and jogging by 313 women (13.3%), whereas aerobics, skiing, swimming, ballgames, and horse riding were each reported by less than 10% of the women.

In addition, 1413 women (60.2%) reported the duration of a typical PA session to be 30 to 60 minutes, whereas 664 women (28.3%) reported <30 minutes and 271 women (11.5% ) 1 to 2 hours. The intensity levels most frequently reported were moderate (1287 [54.8%]), followed by low (876 [37.3%]) and high (186 [7.9%]).

General activity level was estimated for 1430 women (60.9%) reporting moderate- or high-intensity activity. Based on the minimum number of minutes of PA per week (Supplemental Table 1), the median number of active minutes was 120 minutes, with an estimated 386 women (27.0%) performing PA for ≥150 minutes per week (Supplemental Figure 2). Using the maximum estimates (Supplemental Table 1), 711 women (49.7%) were estimated to perform PA of at least 150 minutes per week.

Of the women reporting regular PA before pregancy, 1677 (71.4%) reported less PA during pregnancy, 578 (24.6%) reported similar PA during pregnancy, and 94 (4.0%) reported more PA during pregnancy.

### Secondary aim

For each of the 4 activities most commonly performed at least twice weekly, we calculated that higher PA levels were achieved in 553 women (23.5%) by brisk walking, 287 women (12.2%) by strength training, 263 women (11.2%) by bicycling, and 114 women (4.9%) by jogging.

The results of the univariate analyses of factors associated with higher levels of brisk walking, bicycling, strength training, or jogging are shown in Supplemental Table 2. In the multivariate analysis (Table 2; Figure), regular PA before pregnancy was positively associated with higher levels of brisk walking (odds ratio [OR], 5.30; 95% confidence interval [CI], 3.53–7.97), bicycling (OR, 7.35; 95% CI, 3.59–15.05), and strength training (OR, 10.5; 95% CI, 4.93–22.5). Dog owners were more likely to reach higher levels of brisk walking (OR, 2.18; 95% CI, 1.63–2.91), and those with doctor-diagnosed AD were more likely to reach higher levels of jogging (OR, 2.55; 95% CI, 1.07–6.08) (Figure). The odds for higher levels of brisk walking were reduced in women of non-Nordic origin (OR, 0.53; 95% CI, 0.35–0.81), women living in the suburb (OR, 0.64; 95% CI, 0.45–0.92), and women with previous pregnancy or pregnancies (OR, 0.78; 95% CI, 0.63–0.97). For bicycling, the corresponding reduced odds for higher levels were higher BMI (OR, 0.92; 95% CI, 0.85–0.99), living in the suburb (OR, 0.60; 95% CI, 0.37–0.97), living in the greater Østfold (rural) area (OR, 0.49; 95% CI, 0.25–0.93), and sick leave (OR, 0.50; 95% CI, 0.31–0.81). Higher levels of strength training were less likely in women of non-Nordic origin (OR, 0.44; 95% CI, 0.25–0.78) and in women currently on sick leave (OR, 0.59; 95% CI, 0.39–0.89).

**Table 2:** Results of factors in multivariate analyses that were significantly associated with reaching higher levels of physical activity (≥2 times per week, moderate or high intensity, ≥30 minutes) per week for brisk walking, strength training, bicycling, and jogging

Exposure variable	Brisk walking		Strength training		Bicycling		Jogging	
	OR (95% CI)	*P* value	OR (95% CI)	*P* value	OR (95% CI)	*P* value	OR (95% CI)	*P* value
BMI at 18 wk GA					0.92 (0.85–0.99)	.026^a^		
Study location								
Oslo					Ref	Ref		
Østfold					0.49 (0.25–0.93)	.030^a^		
Sweden					1.25 (0.88–1.78)	.217		
Previous pregnancy or pregnancies								
No	Ref	.026^a^						
Yes	0.78 (0.63–0.97)							
Country of origin								
Norway	Ref	Ref	Ref	Ref				
Sweden	0.93 (0.55–1.56)	.777	0.92 (0.67–1.25)	.583				
Other Nordic countries	1.64 (0.73–3.67)	.229	0.66 (0.20–2.24)	.510				
Rest of the world	0.53 (0.35–0.81)	.003^a^	0.44 (0.25–0.78)	.005^a^				
Living environment								
City center	Ref	Ref			Ref	Ref		
City, outside of city center	0.86 (0.68–1.08)	.193			0.88 (0.65–1.18)	.395		
Suburb	0.64 (0.45–0.92)	.015^a^			0.60 (0.37–0.97)	.038^a^		
Countryside, not in a village	1.08 (0.52–2.24)	.841			0.54 (0.12–2.38)	.414		
Village	0.80 (0.46–1.38)	.420			0.43 (0.15–1.24)	.117		
Regular PA before pregnancy								
No	Ref	Ref	Ref	Ref	Ref	Ref		
Yes	5.30 (3.53–7.97)	<.001^a^	10.50 (4.93–22.50)	<.001^a^	7.35 (3.59–15.05)	<.001^a^		
Dog owner								
No	Ref	Ref						
Yes	2.18 (1.63–2.91)	<.001^a^						
Doctor-diagnosed AD								
No							Ref	Ref
Yes							2.55 (1.07–6.08)	.034^a^
Current sick leave at 18 wk GA								
No			Ref	Ref	Ref	Ref		
Yes			0.59 (0.39–0.89)	.012^a^	0.50 (0.31–0.81)	.005^a^		

The following variables that were significant in univariate analyses but not in multivariate analyses are not shown in the data: age, prepregnancy weight, education, and doctor-diagnosed AR.

*AD*, atopic dermatitis; *BMI*, body mass index; *CI*, confidence interval; *GA*, gestational age; *OR*, odds ratio; *PA*, physical activity; *Ref*, reference.

*Carlsen. Physical activity in midpregnancy. Am J Obstet Gynecol Glob Rep 2020.*

a *P*-values <.05.

**Figure:**
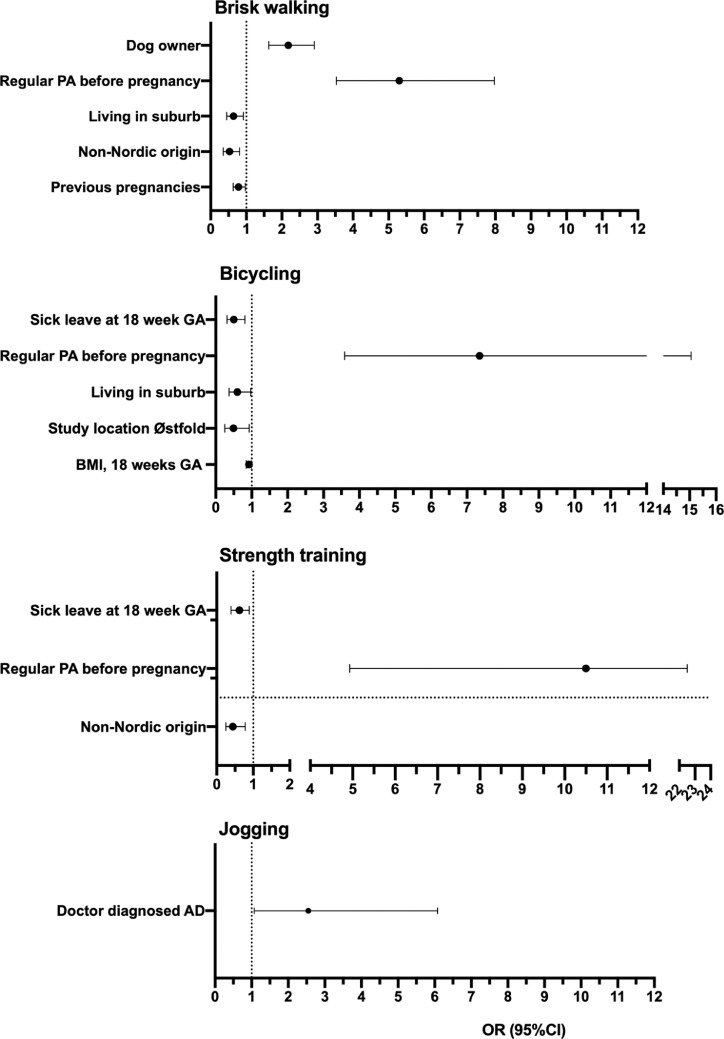
Factors associated with meeting higher levels of PA Factors associated with meeting higher levels of PA by brisk walking (n=553), bicycling (n=263), strength training (n=287), and jogging (n=114) among 2349 pregnant women. Results are shown as ORs with 95% CIs. *AD*, atopic dermatitis; *CI*, confidence interval; *GA*, gestational age; *OR*, odds ratio. Carlsen. Physical activity in midpregnancy. Am J Obstet Gynecol Glob Rep 2021.

## Discussion

### Principal findings

At midpregnancy, 29% of women reported no regular PA. Furthermore, apart from strolling, the most common PA reported at least twice weekly during pregnancy was brisk walking (36% of women), followed by strength training, bicycling, and jogging. The most commonly performed PAs with higher levels in intensity and duration were brisk walking, bicycling, strength training, and jogging. Women reporting regular prepregnancy PA and dog ownership had higher levels of PA, whereas higher BMI, previous pregnancy or pregnancies, non-Nordic origin, living in Østfold county, and being on sick leave were negatively associated with higher levels of PA in pregnancy.

### Results

The frequency of the different activities performed during pregnancy through enrollment in our study is partly in line with other studies. In the Danish National Birth Cohort, including 88,000 pregnancies, approximately one-third of the women reported some type of exercise during early pregnancy or midpregnancy, most often as low-impact activities, such as swimming or bicycling.^27^ Geographic, topographic, and cultural differences may partly explain the differences in preferred PA. For example, bicycling may be favored in countries with flat topography, favorable climatic factors, and traffic facilitation.

Our finding that 13% of women reported strength training at least twice per week was higher than the 8% of women reported in a Danish cross-sectional study of almost 8000 pregnant women in their first trimester of pregnancy^17^ and the 0.3% of women in the ALSPAC study reported at 18 weeks’ gestation.^16^ Jogging at least twice weekly was less common in the PreventADALL study (5%) compared with the Danish study (10%)^17^ but was more common than in the ALSPAC study (0.3%).^16^ Less than 10% of the women in our study reported other PAs performed at least twice weekly, in line with both the Danish and ALSPAC studies.^16^^,^^17^

The higher PA levels observed among 5% (jogging) to 24% (brisk walking) of the women in our study were not directly comparable with studies assessing the proportion of women reaching national recommendations. A Danish study reported that 38% of pregnant women met the Danish guidelines of 3.5 hours of moderate-intensity activity per week^17^ and the ALSPAC study with 49% of women engaging in strenuous exercise at least 3 hours per week.^16^ Our estimates suggested that at least 27% and no more than 50% of women performed 150 minutes or more of PA with moderate or high intensity in our study. This is higher than the 15% of women at 32 weeks’ gestation who performed ≥20 minutes of moderate-intensity activity at least 3 times per week in the Norwegian Akershus Birth Cohort study, which included 3482 women.^18^ This may in part be explained by differences in data collection methods and response categories and, more importantly, by the different duration of pregnancy. Gjestland et al^18^ reported that primiparity, higher education (college or university), and prepregnancy BMI of <30 were associated with increased probability of meeting the national guidelines of 20 minutes of moderate-intensity PA ≥3 times per week.

Women in the PreventADALL study who were physically active before pregnancy were more likely to have higher levels of PA (≥30 minutes ≥2 times per week of at least moderate intensity) during pregnancy, in line with previous studies.^17^^,^^19^ Dog ownership more than doubled the likelihood of higher levels of brisk walking in our study, supported by 50% increased likelihood in the ALSPAC study^23^ and a recent multinational cross-sectional study showing that dog owners walked more and spent more time in outdoor environments.^28^

The Danish cross-sectional study conducted in 2012–2014 identified the following risk factors for not meeting the recommendations of daily PA of 30 minutes at moderate intensity during pregnancy: lack of exercise before pregnancy, being overweight, <4 years of higher education, not being proficient in the Danish language, multiparity, a previous miscarriage, smoking before pregnancy, and becoming pregnant after assisted reproductive technology.^17^

In line with previous studies, higher pregnancy BMI^16^, ^17^, ^18^ and previous pregnancy or pregnancies^16^, ^17^, ^18^, ^19^ were associated with decreased likelihood of higher levels of PA. The reduced likelihood of higher PA levels by women of non-Nordic origin agrees with the Danish findings.^17^ Being on sick leave was associated with reduced likelihood of higher PA levels by strength training and bicycling.

In contrast to other studies,^16^, ^17^, ^18^, ^19^^,^^27^ neither education nor age was significantly associated with higher levels of the 4 most commonly performed PAs. However, these findings were supported by a Portuguese study, including 133 women during the first 2 trimesters of pregnancy.^29^ Our cohort was somewhat biased in terms of education, as more than 50% of the women had ≥4 years of higher education; however, the women in the previous Norwegian,^18^ Danish,^17^ and ALSPAC^16^ studies had similar educational levels.

Higher levels of PA were not significantly associated with doctor-diagnosed asthma or AR. This may suggest that mild or well-regulated allergic disease does not limit PA. However, we did find that doctor-diagnosed AD was positively associated with higher levels of jogging. To the best of our knowledge, this is a novel finding with unclear implications. A recent study from the United States^30^ found that AD was associated with less PA in US adults, whereas a systematic review from 2016^31^ found insufficient evidence to conclude whether AD was associated with more or less PA. In addition, we are not aware of any previous studies reporting higher levels of jogging in women with AD.

### Strengths and limitations

This study offered study participation to all pregnant women at 16 to 22 weeks’ gestation who attended the national routine fetal ultrasound screening in their midtrimester of pregnancy. Unintentionally, the enrolled study population had higher education attainment, had slightly higher age than the national average, and was predominantly of Norwegian and Swedish origins but is relatively representative of city populations.^32^^,^^33^ Furthermore, our population matched that of other similar cohort studies in terms of age, parity, education, and income levels.^17^^,^^18^ The skewing of our population toward higher education may have bearings on the generalizability of our results, with PA observed in our study possibly overestimating that of the general population.

However, if our finding that 29% of the women had been inactive at midpregnancy is an overrepresentation, the general population may be even less active than the population in the PreventADALL study.

A limitation in the PreventADALL study was that women without sufficient Norwegian or Swedish language skills were excluded from participation. Therefore, our study is not generalizable for some minority populations. Furthermore, the questionnaire was not appropriate for direct comparison with the current Norwegian guidelines published in April 2019. The information on PA in pregnancy included questions in line with those reported by Haakstad et al^25^ in a Norwegian pregnancy cohort but were modified to fit our electronic questionnaire. Because of the study design, it was not feasible to include accelerometer or other objective measures of PA; therefore, the data presented in this article were exclusively self-reported. Contraindications for PA were not explored in this study, as the study population consisted of relatively healthy women, pregnant with 1 or 2 fetuses.

### Clinical implications

Despite the acknowledged benefits to maternal and offspring health by regular PA in pregnancy, our data showed that less than 50% of the women were regularly active at a high level in midpregnancy. This pointed to a need to address the importance of PA during pregnancy follow-up visits.

### Research implications

The potential benefits of high levels of PA in pregnancy for the mother and her offspring in terms of noncommunicable disease development need further investigations, as do the potential effects of suboptimal levels of PA in pregnant women.

## Conclusion

At midpregnancy, almost one-third of women reported no regular PA in the last 2 weeks before answering the questionnaire, whereas less than 50% of women had 2 hours or more of regular moderate-intensity PA per week during pregnancy. The most common activities performed at least twice weekly were brisk walking, bicycling, and strength training. Being physically active before pregnancy, owning a dog, and having AD were associated with higher levels of 1 or more of the most commonly performed physical activities. Awareness of PA in pregnancy should be discussed at pregnancy follow-up visits, particularly among women with higher BMI, on sick leave, with previous pregnancy or pregnancies, and of non-Nordic origin—groups who often do not reach higher levels of PA.

## Appendix Supplementary materials

Image, application 1
