# Supplementary material for: Physical activity in pregnancy: a Norwegian-Swedish mother-child birth cohort study
Source: AJOG Glob Rep. 2021 Jan 27;1(1):100002. doi: 10.1016/j.xagr.2020.100002 (PMC9563683; doi:10.1016/j.xagr.2020.100002)
Supplement: Supplementary file 1 [file mmc1.pdf]

Supplemental Information

Methods

Body mass index (BMI) and weight at 18 weeks' gestation are closely correlated variables; the same applies for previous pregnancy or pregnancies and previous delivery or deliveries. We used BMI and previous pregnancy or pregnancies in our final multivariable model.

We chose the variable previous pregnancy or pregnancies instead of previous delivery of deliveries in our final multivariate model, as previous miscarriage or miscarriages may affect levels of physical activity (PA) in the current pregnancy.[31]

Calculations of minutes of physical activity per week, minimum and maximum

Minimum

The minimum number of sessions multiplied by the minimum duration in minutes, given that the intensity is at least moderate.

Maximum

The maximum number of sessions multiplied by the maximum duration in minutes, given that the intensity is at least moderate.

All activities except strolling are included in these calculations, and all number of sessions are included except rarely or never.

It was not possible to calculate the exact number of minutes of physical activity, as the women were only asked to report the usual duration of a typical session of physical activity and not the duration of each type of activity or how many sessions they had in a typical week. In addition, many (but not all) alternatives in the electronic questionnaire had a range, both for the number of sessions

and for the usual duration of typical session. The alternatives for the duration and number of sessions are outlined in Supplemental Table 1, providing the basis for the estimated weekly PA duration.

Supplemental Figure 1 Frequencies of physical activities

The frequencies of physical activities performed at least once per week during the first 18 weeks of pregnancy that are shown are based on 2349 women from the Preventing Atopic Dermatitis and Allergies in Children study. Activities reported performed less than once per week are not shown in the graph; n represents the number of women participating in the respective activity during pregnancy.

<FIGSE>*Carlsen. Physical activity in midpregnancy. Am J Obstet Gynecol Glob Rep 2020.*

Supplemental Figure 2 Histogram of the minimum number of minutes of physical activity

The histogram shows the minimum number of minutes of physical activity per week. Only women (n=1430) reporting moderate- or high-intensity activities, other than strolling, are included. The reference lines on the x-axis represent 120 minutes per week (*solid line*) and 150 minutes per week (*dashed line*).

<FIGSE>*Carlsen. Physical activity in midpregnancy. Am J Obstet Gynecol Glob Rep 2020.*

Supplemental Table 1 The usual duration of a physical activity session in minutes and the number of sessions per week

Duration		
Categories in the e-questionnaire	Min used to calculate the minimum number of min	Min used to calculate the maximum number of min
<30 min	1	29
30–60 min	30	60
1–2 h	60	120
>2 h	121	121

Sessions per wk		
Categories in the e-questionnaire	Sessions used to calculate the minimum number of min	Sessions used to calculate the maximum number of min
Rarely or never	0.00	0.00
1–3 per mo	0.25	0.75
1 per wk	1.00	1.00
2–3 per wk	2.00	3.00
4–5 per wk	4.00	5.00
5–6 per wk	5.00	6.00
1 per d	7.00	7.00
>1 per d	8.00	8.00

The e-questionnaire categories were recoded into numbers to calculate the minimum and maximum number of minutes per week. Because of the phrasing of questions, we were unable to directly derive the proportion of women who had at least 150 minutes regular PA per week of moderate or high

intensity. To account for the uncertainties in our estimates of reaching this PA level, we calculated the minimum and maximum possible numbers of minutes, including all reported activities and durations for women reporting moderate- or high-intensity PA. The “true” proportion of women with high PA value is somewhere between the upper and lower time limits of each category. Women reporting combined sessions had 1 session registered for each of the activities performed during the session. For example, a session of jogging of 10 to 15 minutes (warm-up), strength training of 45 minutes, and yoga of 15 minutes (stretching), with a typical duration of 1 to 2 hours, should correctly be defined as a single session of 70 to 75 minutes, but with our method, this may have been counted as 3 sessions of 1 to 2 hours each. Together with no upper limit on the highest categories, this left us unable to estimate the mean number of minutes.

e-questionnaire, electronic questionnaire.

Carlsen. Physical activity in midpregnancy. Am J Obstet Gynecol Glob Rep 2020.

Supplemental Table 2 Univariate logistic regression analyses exploring potential factors associated with higher levels (≥2–3 times per week, moderate or intensity, ≥30 minutes) of brisk walking, strength training, bicycling, and jogging

Higher level of PA	Brisk walking		Strength training		Bicycling		Jogging	
Yes	n=553		n=287		n=263		n=114	
No	n=1796		n=2062		n=2086		n=2235	
	OR (95% CI)	*P* value (global and categorical)	OR (95% CI)	*P* value (global and categorical)	OR (95% CI)	*P* value (global and categorical)	OR (95% CI)	*P* value (global and categorical)
Age categories (y)		.206		.012[a]		.285		.533
20–29	Ref	Ref	Ref	Ref	Ref	Ref	Ref	Ref
30–31	0.88 (0.66–1.16)	.363	1.27 (0.89–1.80)	.187	1.50 (1.01–2.22)	.044	0.96 (0.55–1.66)	.882
		.057		.882		.155		.976

32–33<br>34–36<br>37–48	0.75 (0.56–1.01)<br>0.78 (0.59–1.03)<br>0.65 (0.55–1.01)	.076<br>.057	0.96 (0.66–1.40)<br>0.85 (0.59–1.23)<br>0.59 (0.38–0.92)	.391<br>.020[a]	1.34 (0.89–2.02)<br>1.32 (0.89–1.96)<br>1.11 (0.72–1.72)	.167<br>.633	1.01 (0.58–1.74)<br>0.76 (0.43–1.33)<br>0.63 (0.33–1.21)	.332<br>.164
Age continuous variable	0.98 (0.95–1.00)	.048[a]	0.97 (0.94–1.00)	.029[a]	1.01 (0.98–1.04)	.452	0.98 (0.93–1.02)	.297
Prepregnancy weight	0.99 (0.98–1.00)	.019[a]	0.99 (0.98–1.00)	.249	0.98 (0.97–1.00)	.012[a]	0.97 (0.95–0.99)	.001[a]
Weight gain at 18 wk GA	1.01 (0.98–1.04)	.398	1.03 (0.99–1.07)	.173	0.99 (0.95–1.03)	.539	1.02 (0.96–1.08)	.493

Weight at 18 wk GA	0.99 (0.98–1.00)	.035[a]	1.00 (0.98–1.00)	.410	0.98 (0.97–1.00)	.011[a]	0.97 (0.95–0.99)	.002[a]
BMI at 18 wk GA	0.95 (0.92–0.98)	<.001[a]	0.97 (0.94–1.00)	.136	0.93 (0.90–0.97)	.001[a]	0.89 (0.84–0.95)	.001[a]
Study location		.005[a]		.461		<.001[a]		.506
Oslo	Ref		Ref		Ref	Ref	Ref	Ref
Østfold	0.69 (0.51–0.92)	.013[a]	1.05 (0.73–1.49)	.804	0.28 (0.16–0.51)	<.001[a]	0.80 (0.45–1.44)	.461
Sweden	0.74 (0.58–0.94)	.013[a]	0.83 (0.61–1.14)	.261	0.91 (0.67–1.24)	.556	0.78 (0.48–1.27)	.314
Marital status		.105		.224		.356		.792
Married or cohabitant	Ref	Ref	Ref	Ref	Ref	Ref	Ref	Ref

Single	0.82 (0.39–1.73)	.609	0.51 (0.16–1.67)	.269	1.50 (0.66–3.39)	.335	0.94 (0.22–3.93)	.932
Other	0.23 (0.07–1.19)	.084	1		1		1.72 (0.40–7.37)	.468
Previous pregnancy or pregnancies		<.001[a]		<.001[a]		.097		.015[a]
No	Ref	Ref	Ref	Ref	Ref	Ref	Ref	Ref
Yes	0.63 (0.52–0.76)	<.001[a]	0.61 (0.48–0.78)	<.001[a]	0.80 (0.62–1.04)	.096	0.62 (0.43–0.91)	.015[a]
Previous delivery or deliveries		<.001[a]		<.001[a]		.386		.031[a]
0	Ref	Ref	Ref	Ref	Ref	Ref	Ref	Ref

1	0.58 (0.46–0.72)	<.001[a]	0.53 (0.39–0.71)	<.001[a]	0.86 (0.65–1.14)	.301	0.55 (0.35–0.88)	.012[a]
≥2	0.59 (0.40–0.86)	.006[a]	0.41 (0.23–0.73)	.003[a]	0.76 (0.46–1.26)	.289	0.80 (0.40–1.62)	.537
Education		.001[a]		.941		<.001[a]		.179
Primary school only	Ref	Ref	Ref	Ref	Ref	Ref	Ref	Ref
High school	0.55 (0.19–1.60)	.273	0.82 (0.37–1.84)	.632	0.92 (0.11–7.49)	.935	0.41 (0.05–3.65)	.421
University <4 y	0.80 (0.29–2.25)	.677	0.94 (0.45–1.95)	.860	1.60 (0.21–12.2)	.647	0.92 (0.12–7.08)	.937
University >4 y	1.08 (0.39–2.99)	.887	0.91 (0.44–1.87)	.790	3.03 (0.40–22.80)	.281	1.10 (0.15–8.36)	.924
	1.02 (0.32–				6.98 (0.87–		1.21 (0.13–	

PhD	3.23)	.973	1		55.99)	.067[a]	11.5)	.870
Family income		.205		.547		.011[a]		.157
<300,000 NOK/SEK	Ref	Ref	Ref	Ref	Ref	Ref	Ref	Ref
300,000–600,000 NOK/SEK	0.96 (0.39–2.32)	.922	1.93 (0.44–8.42)	.384	0.91 (0.26–3.18)	.878	0.80 (0.17–3.69)	.771
600,000–1,000,000 NOK/SEK	0.97 (0.41–2.28)	.938	2.02 (0.48–8.59)	.340	0.97 (0.29–3.25)	.956	0.80 (0.19–3.46)	.770
1,000,000–1,400,000 NOK/SEK	1.11 (0.47–2.62)	.820	2.03 (0.48–8.65)	.340	1.34 (0.40–4.49)	.640	1.17 (0.27–5.00)	.834
>1,400,000 NOK/SEK	1.11 (0.45–2.69)	.824	1.82 (0.41–8.00)	.430	1.66 (0.48–5.71)	.423	1.56 (0.35–6.94)	.559
Do not wish to answer	0.36 (0.09–		0.72 (0.10–		0.23 (0.02–		1	

	1.35)	.128	5.41)	.748	2.23)	.207		
Country of origin		<.001[a]		.005[a]		.257		.290
Norway	Ref	Ref	Ref	Ref	Ref	Ref	Ref	Ref
Sweden	0.77 (0.61–0.98)	.035[a]	0.83 (0.61–1.13)	.245	1.03 (0.76–1.41)	.835	0.69 (0.42–1.14)	.145
Other Nordic countries	1.38 (0.65–2.96)	.404	0.69 (0.21–2.29)	.544	2.33 (0.99–5.48)	.053	0.58 (0.08–4.30)	.593
Rest of the world	0.48 (0.33–0.70)	<.001[a]	0.41 (0.24–0.72)	.002[a]	0.83 (0.52–1.33)	.439	0.62 (0.30–1.29)	.201
Living environment		.002[a]		.417		<.001[a]		.314
City center	Ref	Ref	Ref	Ref	Ref	Ref	Ref	Ref
City, outside of city center	0.82 (0.66–	.067[a]	0.93 (0.70–	.586	0.75 (0.57–	.042[a]	0.89 (0.58–	.573

	1.01)		1.22)		0.99)		1.35)	
Suburb	0.56 (0.41–0.76)	<.001[a]	0.74 (0.50–1.08)	.122	0.49 (0.32–0.74)	.001[a]	0.53 (0.27–1.02)	.058
Countryside, not in a village	0.90 (0.47–1.72)	.752	0.69 (0.27–1.77)	.442	0.24 (0.06–1.00)	.049[a]	0.69 (0.16–2.93)	.671
Village	0.63 (0.39–1.01)	.053[a]	0.68 (0.36–1.27)	.223	0.19 (0.07–0.54)	.002[a]	1.16 (0.54–2.51)	.701
Regular PA before pregnancy		<.001[a]		<.001[a]		<.001[a]		
No								
Yes	Ref	Ref	Ref	Ref	Ref	Ref	Ref	
	5.76 (3.90–8.50)	<.001[a]	11.36 (5.33–24.2)	<.001[a]	7.85 (4.01–15.4)	<.001[a]	1	

Dog owner		<.001		.213		.141		.315
No	Ref	Ref	Ref	Ref	Ref	Ref	Ref	Ref
Yes	1.96 (1.51–2.54)	<.001[a]	1.25 (0.88–1.78)	.204	0.73 (0.48–1.12)	.155	1.31 (0.78–2.21)	.302
Cat owner		.997		.069		.082		.624
No	Ref	Ref	Ref	Ref	Ref	Ref	Ref	Ref
Yes	1.00 (0.74–1.36)	.997	1.41 (0.98–2.02)	.061	0.67 (0.42–1.07)	.097	0.86 (0.45–1.62)	.631
Current sick leave		.015[a]		.005[a]		<.001[a]		.029[a]
No	Ref	Ref	Ref	Ref	Ref	Ref	Ref	Ref
Yes	0.71 (0.54–0.94)	.018[a]	0.58 (0.39–0.87)	.008[a]	0.44 (0.23–0.70)	<.001[a]	0.51 (0.26–0.99)	.046[a]

Doctor-diagnosed asthma		.218		.931		.559		.930
No	Ref	Ref	Ref	Ref	Ref	Ref	Ref	Ref
Yes	1.17 (0.91–1.49)	.214	1.01 (0.73–1.41)	.931	0.90 (0.64–1.28)	.562	1.02 (0.62–1.68)	.930
Doctor-diagnosed AD		.954		.381		.786		.033[a]
No	Ref	Ref	Ref	Ref	Ref	Ref	Ref	Ref
Yes	0.99 (0.70–1.39)	.954	1.22 (0.78–1.93)	.384	0.94 (0.58–1.50)	.786	2.37 (1.01–5.52)	.047[a]
Doctor-diagnosed allergic rhinitis		.655		.720		.796		.041[a]
No	Ref	Ref	Ref	Ref	Ref	Ref	Ref	Ref
Yes	1.06 (0.83–		0.94 (0.69–		1.04 (0.76–		0.58 (0.33–	

	1.33)	.654	1.29)	.721	1.43)	.759	1.01)	.054[a]
Smoking in pregnancy		.528		.300		.518		.967
Not in pregnancy	Ref	Ref	Ref	Ref	Ref	Ref	Ref	Ref
Quit before 18 wk GA	0.96 (0.57–1.62)	.874	1.24 (0.66–2.32)	.018	0.71 (0.32–1.55)	.385	1.00 (0.36–2.80)	.994
Smoking at 18 wk GA	0.46 (0.10–2.04)	.307	2.44 (0.78–7.64)	.124	0.51 (0.07–3.87)	.517	1.32 (0.17–10.11)	.788
Snus in pregnancy		.242		.908		.163[a]		.085[a]
Not in pregnancy	Ref	Ref	Ref	Ref	Ref	Ref	Ref	Ref
Quit before 18 wk GA	1.24 (0.87–1.78)	.231	0.93 (0.57–1.53)	.780	1.54 (0.99–2.39)	.054[a]	1.75 (0.96–3.20)	.067[a]
	2.07 (0.68–		1.30 (0.29–		1.50 (0.33–		1	

Snus at 18 wk GA	6.36)	.203	5.91)	.732	6.80)	.601		

AD, atopic dermatitis; *BMI*, body mass index; *CI*, confidence interval; *GA*, gestational age; *NOK*, Norwegian Krone; *OR*, odds ratio; *PA*, physical activity; *Ref*, reference; *SEK*, Swedish Krona.

Carlsen. Physical activity in midpregnancy. Am J Obstet Gynecol Glob Rep 2020.
